# Metabolomic comparison followed by cross-validation of enzyme-linked immunosorbent assay to reveal potential biomarkers of diabetic retinopathy in Chinese with type 2 diabetes

**DOI:** 10.3389/fendo.2022.986303

**Published:** 2022-09-08

**Authors:** Zongyi Wang, Jiyang Tang, Enzhong Jin, Chi Ren, Siying Li, Linqi Zhang, Yusheng Zhong, Yu Cao, Jianmin Wang, Wei Zhou, Mingwei Zhao, Lvzhen Huang, Jinfeng Qu

**Affiliations:** ^1^ Department of Ophthalmology, Peking University People’s Hospital, Eye Diseases and Optometry Institute, Beijing Key Laboratory of Diagnosis and Therapy of Retinal and Choroid Diseases, College of Optometry, Peking University Health Science Center, Beijing, China; ^2^ Department of Ophthalmology, The Second Hospital of Hebei Medical University, Shijiazhuang, China; ^3^ Department of Ophthalmology, Tianjin Medical University General Hospital, Tianjin, China

**Keywords:** biomarker, diabetic retinopathy, enzyme-linked immunosorbent assay, metabolomics, non-proliferative diabetic retinopathy, proliferative diabetic retinopathy, type 2 diabetes, targeted metabolomics

## Abstract

**Purpose:**

To identify the biomarkers in the critical period of development in diabetic retinopathy (DR) in Chinese with type 2 diabetes using targeted and untargeted metabolomics, and to explore the feasibility of their clinical application

**Methods:**

This case-control study described the differential metabolites between 83 Chinese type 2 diabetes mellitus (T2DM) samples with disease duration ≥ 10 years and 27 controls matched cases. Targeted metabolomics using high-resolution mass spectrometry with liquid chromatography was performed on plasma samples of subjects. The results were compared to our previous untargeted metabolomics study and ELISA was performed to validate the mutual differential metabolites of targeted and untargeted metabolomics on plasma. Multiple linear regression analyses were performed to adjust for the significance of different metabolites between groups.

**Result:**

Mean age of the subjects was 66.3 years and mean T2DM duration was 16.5 years. By cross-validating with results from previous untargeted metabolomic assays, we found that L-Citrulline (Cit), indoleacetic acid (IAA), 1-methylhistidine (1-MH), phosphatidylcholines (PCs), hexanoylcarnitine, chenodeoxycholic acid (CDCA) and eicosapentaenoic acid (EPA) were the most distinctive metabolites biomarkers to distinguish the severity of DR for two different metabolomic approaches in our study. We mainly found that samples in the DR stage showed lower serum level of Cit and higher serum level of IAA compared with samples in the T2DM stage, while during the progression of diabetic retinopathy, the serum levels of CDCA and EPA in PDR stage were significantly lower than NPDR stage. Among them, 4 differential key metabolites including Cit, IAA, CDCA and EPA were confirmed with ELISA.

**Conclusion:**

This is the first study to compare the results of targeted and untargeted metabolomics *via* liquid chromatography-mass spectrometry to find the serum biomarkers which could reflect the metabolic variations among different stages of DR in Chinese. The progression of DR in Chinese at different critical stages was related to the serum levels of specific differential metabolites, of which there is a significant correlation between DR progression and increased IAA and decreased Cit, hexanoylcarnitine, CDCA, and EPA. However, larger studies are needed to confirm our results. Based on this study, it could be inferred that the accuracy of targeted metabolomics for metabolite expression in serum is to some extent higher than that of untargeted metabolomics.

## 1 Introduction

Diabetic retinopathy (DR) as a destructive disease is the most serious microvascular complication of diabetes in eyes (1–3) and the main cause of hypopsia and blindness among 20 to 74 year-old adults in developing and developed countries (4–6). A study showed that China had 114 million diabetics, ranking first in the world (7, 8). In China, the prevalence of DR in the general population was 1.7%, while the prevalence of DR in the diabetic population was 22.4%, with the greatest prevalence in North China (27.7%) (8). Currently, the treatments of DR, including retinal laser photocoagulation, intravitreal injection of anti-vascular endothelial growth factor and vitrectomy are only aimed at controlling the late development of DR, and there is no effective treatment to limit neurovascular dysfunction or promote repair in the early stages of DR (9). In addition, for a long time, the blood glucose level and duration of diabetes have been considered to be the main risk factors for the development of DR (2, 10). However, in clinical practice, these risk factors cannot well explain the huge difference in the rate of individual progression of DR (11, 12) which indicates that there may be other unknown factors that can better screen and predict the occurrence and development of DR.

Although many metabolomic studies of DR have been conducted, the identification of differential metabolites in critical periods of DR development (periods of T2DM and NPDR) has been rarely attempted, especially in Chinese populations. In our previous untargeted metabolomics study of DR in Chinese, we found that in addition to the dysregulation of the classic amino acid metabolic pathway, many small molecules such as long-chain polyunsaturated fatty acids, phosphatidylcholines (PCs) and bile acids were up- or down-regulated to varying degrees during the critical periods of DR (13). The main purpose of untargeted metabolomics is to discover the metabolites in the sample as many as possible and reflect the information of total metabolites to the greatest extent, which helps to discover the unknown key metabolites. Targeted metabolomics uses target compound standards as a reference to detect and analyze specific metabolites in biological samples in a targeted manner, which can more accurately identify the target metabolites (14, 15).

To our best knowledge, there have been no studies using the same detection platform to compare the untargeted and targeted metabolomic outcomes in different stages of DR samples. To fill this gap, this study aimed to perform targeted metabolomics via liquid chromatography-mass spectrometry (LC-MS) in serum of the T2DM Chinese with and without DR. And the results of the targeted metabolomics were compared with those of previous untargeted metabolomics to identify the biomarkers which have a positive or negative impact on the development of DR and are associated with DR prognosis. In addition, we further used ELISA to revalidate these differential metabolites critical to the course of DR.

## 2 Methods

### 2.1 Study participants and study design

**Figure 1:**
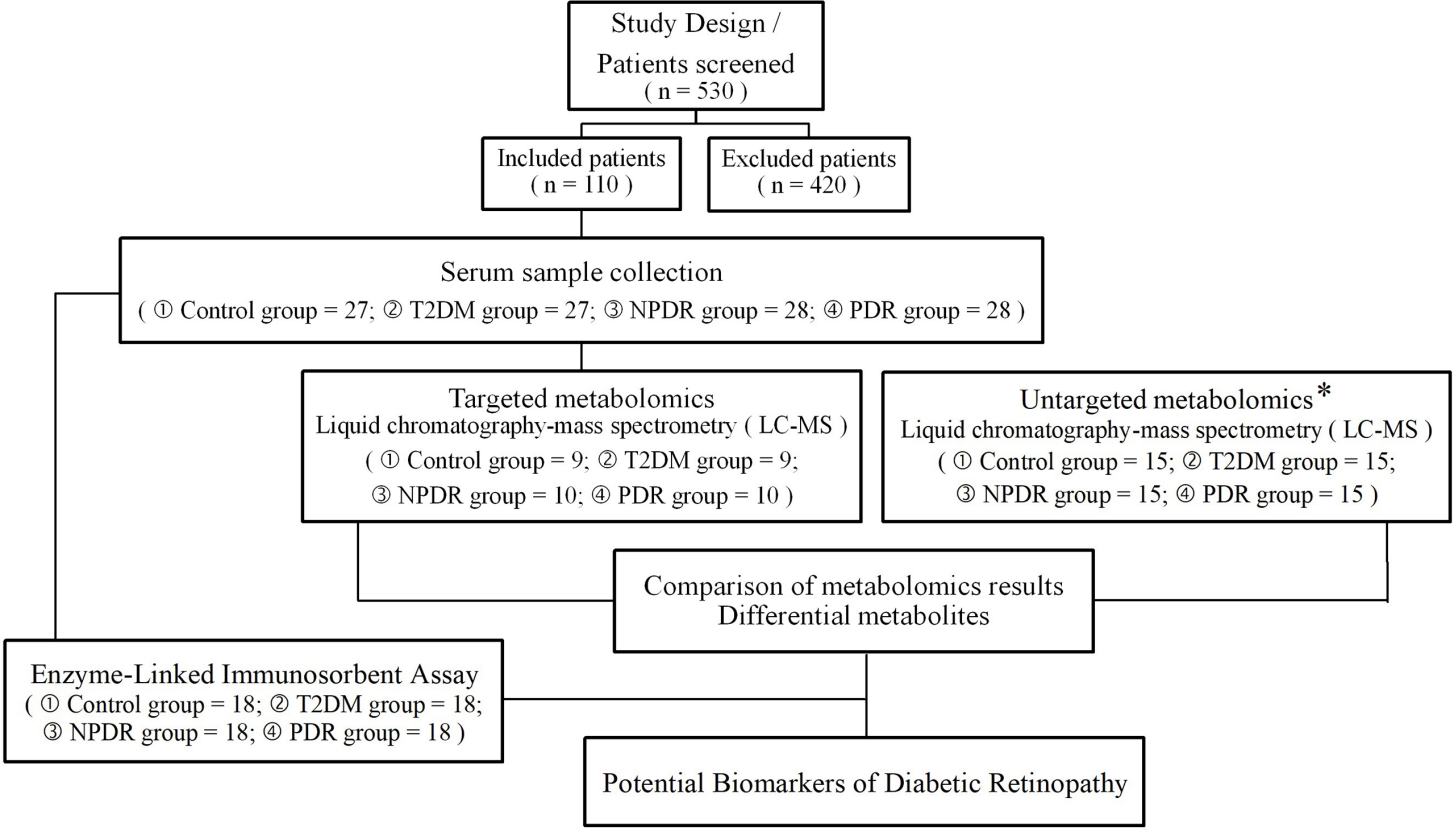
An overview of the metabolomics analysis workflow, and the inclusion and exclusion flowchart of the case-control study. * The sample collection and testing in untargeted metabolomics were conducted in our previous study. T2DM, type 2 diabetes mellitus; DR, diabetic retinopathy; NPDR, non-proliferative diabetic retinopathy; PDR, proliferative diabetic retinopathy.

We conducted this case-control study, which was registered on May 13th, 2022, and included diabetic patients at Peking University People’s Hospital Ophthalmologic Center from June 1st, 2021, to May 1st 2022. A total of 530 samples with type 2 diabetes were screened and a cohort of 110 samples was recruited. This case-control study was approved by the Ethical Committee of Peking University People’s Hospital (Approval Number: 2021PHB112-001). This research adhered to the tenets of the Declaration of Helsinki. Written informed consent was obtained from all participants prior to study enrollment. To match clinical parameters between case and control subjects, the control subjects (n = 27) were healthy individuals, the T2DM group (n = 27) included samples with a diagnosis of type 2 diabetes for at least 10 years with no clinical signs of DR, while DR cases including NPDR group (n = 28) and PDR group (n = 28) were type 2 diabetes samples with clinical signs of DR. In this study, the control group (n = 27), T2DM group (n = 27), NPDR group (n = 28) and PDR group (n = 28) were respectively and randomly divided into 9 control, 9 T2DM, 10 NPDR and 10 PDR samples for targeted metabolomics research and the other samples included control group (n = 18), T2DM group (n = 18), NPDR group (n = 18) and PDR group (n = 18) were conducted for ELISA test (**Figure 1**).

### 2.2 Diabetic retinopathy phenotyping

In accordance with Early Treatment Diabetic Retinopathy Study (ETDRS) criteria, DR was graded into three categories: no DR, NPDR or PDR (16, 17). All participants were diagnosed upon dilated fundus examination by two retina specialists. Presence of DR was confirmed and documented with color fundus photography, fluorescein angiography (FA) and optical coherence tomography (OCT), classifying study eyes as NPDR (n = 28) or PDR (n = 28) eyes. Color fundus photography and fluorescein angiography (FA) were obtained with FF 540 Plus (Carl Zeiss Meditech, Jena, Germany) or Optos 200Tx (Optos plc, Dunfermline, Scotland, UK). Optical coherence tomography (OCT) was performed with RTVue XR Avanti (Optovue, Fremont, CA, USA) or Cirrus HD-OCT 5000 (Carl Zeiss Meditec Inc, Dublin, CA, USA). Two or more ophthalmologists evaluated the DR status based on the results of the exams to avoid potential diagnosis bias. If there was discordance between the evaluators, they reviewed the images and agreed on the final interpretation. Participants with following situation would be excluded: (1) presence or history of other eye diseases (retinal degeneration, glaucoma, active ocular inflammation etc.); or history of intraocular surgery (vitreoretinal surgery, intravitreal injection of anti- vascular endothelial growth factor (VEGF) or other drugs, laser therapy) or trauma; (2) cancer, infectious disease, hyperuricemia, inherited metabolic diseases, mental disorder, heart failure, severe hypertension, acute myocardial infarction, stroke or any other severe chronic systemic disease; (3) corneal and lens pathologies that prevent a clear view of the fundus. Only those following none of the exclusion criteria for both cases and controls were potential participants.

### 2.3 Data collection and definitions

All of the participants’ medical history and relevant personal history, including age, sex, duration of DM, past medical history, current status of smoking and alcohol consumption, duration of diabetes, treatment history, clinical and laboratory measurements, medication history and disease status were obtained. All participants underwent a physical examination. Blood pressure and body mass index (BMI) measurements were recorded. Blood laboratory tests taken on the closest date (within 3 days) to blood draw including fasting plasma glucose (FPG), total cholesterol (TC), triglycerides (TG), high density lipoprotein cholesterol (HDL-c), low density lipoprotein cholesterol (LDL-c), serum creatinine (SCr), hemoglobin A1c (HbA1c), and blood urea nitrogen (BUN) were measured using standard automated assays and were recorded in the electronic case report form.

### 2.4 Sample preparation for metabolomic study

After at least 8 hours of overnight fasting, 6 mL of venous blood samples were collected under complete aseptic precautions from each study participant with tubes and stored at 4°C. The serum was separated by centrifugation at 3000 rpm for 10 min (4°C) within 30 minutes to separate plasma from whole blood, then the plasma was transferred into a 1.5 mL sterile tube and stored at −80°C ultra- low temperature freeze immediately. Well-trained professional technicians would then carry out further measurements.

### 2.5 Targeted metabolomics analysis

Targeted quantitative metabolomics analysis was performed on the Biocrates P500 platform using the MxP500 Quant kit (Biocrates Life Science AG, Innsbruck, Austria). Thawed frozen plasma samples (10 μL) were transferred to a 56-well plate, dried under a nitrogen stream and added 5% phenylisothiocyanate (PITC) solution for derivatization. After 1 hour of incubation in the dark, the samples were dried for two hours under nitrogen stream. The filtered extracts (obtained before adding 300 μl of extraction solvent and mixing at 450 rpm for 30 min) were collected by centrifugation at 600 rpm for 10 minutes for subsequent analysis after further dilution.

Metabolites which were extracted on a MetLMS system (Biocrates Life Science AG, Innsbruck, Austria) were analyzed by liquid chromatography-tandem mass spectrometry (LC–MS/MS) and flow injection analysis-tandem mass spectrometry (FIA-MS/MS) using multiple reaction monitoring to detect the analytes. Five microlitres of diluted sample extract was used for the LC-MS/MS in positive and negative mode and injected onto a Biocrates® MxP® Quant 500 UHPLC column (Biocrates® Part No.: 22005) at 50 °C using solvent A (water containing 0.2% formic acid) and solvent B (acetonitrile containing 0.2% formic acid). For the FIA-MS/MS, twenty microlitres of the diluted sample extract (diluted in the Biocrates MS Running Solvent) was used for flow injection analysis via tandem-mass spectrometry (FIA-MS/MS) acquisition in positive mode. LC–MS/MS and FIA-MS/MS analysis were performed using a SCIEX Triple QuadTM 6500+ system (Sciex, Darmstadt, Germany) and Acquity H-Class ultra-high performance liquid chromatograph system (Waters).

### 2.6 Untargeted metabolomics analysis

In our previous untargeted metabolomics study (13), we used Vanquish UHPLC system (ThermoFisher, Germany) with an Orbitrap Q ExactiveTM HF mass spectrometer (Thermo Fisher, Germany) for untargeted metabolomic analysis. The raw data files generated by UHPLC-MS/MS (Ultra High-Performance Liquid Chromatography coupled to Tandem Mass Spectrometry) were processed using the Compound Discoverer 3.1 (CD3.1, Thermo Fisher) to perform peak alignment, peak picking, and quantitation for each metabolite. Normalized data was used to predict molecular formulas based on additive ions, molecular ion peaks, and fragment ions. Peaks were matched with the mzCloud, mzVault and MassList database for accurate qualitative and relative quantitative results. Statistical analysis was performed using the statistical software R (R version R-3.4.3), Python (Python 2.7.6 version) and CentOS (CentOS version 6.6). As with targeted metabolomics, P < 0.05 was considered statistically significant. Fold change (FC) ratios > 1.2 and < 0.833 were used to indicate significantly up- and down-regulated differential metabolites, respectively. Detailed information is presented in the GitHub page (https://github.com/zoe19930939/metabolomic2022.github.io.git). Through untargeted metabolomics, we compared the differential metabolites that met the above conditions with the differential metabolites of targeted metabolomics to determine the key metabolites that appeared in both targeted and untargeted metabolomics.

### 2.7 The detection method of ELISA

The level of Cit, EPA and IAA for each sample were measured using an ELISA kit (CEA505Ge, CEO122Ge and CEA737Ge, Wuhan CLOUD-CLONE CORP. technology Co., Ltd., China). And the level of CDCA for each sample was measured using an ELISA kit (MET-5008, Cell Biolabs Inc., San Diego, USA). Manufactures instructions were followed for each kit.

### 2.8 Statistical analysis

Descriptive statistics for demographic and clinical variables of study population were used. Analysis of variance (ANOVA) was used to compare means of normally distributed data with homogeneity of variances. Chi-square test was used for analysis of categoric data (e.g., gender and presence of comorbidities). Wilcoxon rank sum test was performed to compare age, diabetes duration and biochemical parameters. Multiple linear regression was adopted to analyze the differential metabolites between groups and introduce dummy variable to analyze the influence of groups on dependent variables. P-value < 0.05 was considered statistically significant.

The raw data from targeted metabolomics analysis were analyzed in MetILMS version Oxygent-DB110-3005 (Biocrates Life Science AG, Innsbruck, Austria). R statistical software (vision 3.5.2) was used for statistical analysis and visualization of the results. P < 0.05 was considered as statistically significant. Fold change (FC) ratios > 1.2 were considered to indicate up-regulation, and FC ratios < 0.833 were considered to indicate down-regulation. Orthogonal partial least squares- discriminant analysis (OPLS-DA), a volcano map and heat map were used as complementary approaches to identify metabolic features that distinguish different stages of DR samples from controls. Receiver operating characteristic (ROC) curve analysis indicated that the area under the ROC curve (AUC), 95% CI and the AUC ≥0.8 were considered good assessments of the utility of a biomarker.

For detailed information, please refer to: https://github.com/zoe19930939/metabonomic2022.github.io.git.

## 3 Result

### 3.1 Baseline characteristics

Of the 123 subjects recruited in the study, clinical data and samples were collected from 110 subjects who gave consent and completed ophthalmologic exams. The mean age of the participants was 66.3 years, the median duration of diabetes mellitus was 16.5 years, and 47.4% of all participants were females. Among a total of 110 participants who underwent ophthalmologic assessment, 27 were T2DM samples with no sign of DR (mean age of 65.75 ± 7.64 years, 39.3% males), 28 were NPDR samples (mean age of 68.72 ± 9.31 years, 69.0% males), 28 were PDR samples (mean age of 63.59 ± 6.97 years, 55.2% males) and 27 were controls (mean age of 67.18 ± 7.77 years, 46.4% males). Samples and controls with no significant differences in clinical characteristics except for blood urea nitrogen and the presence or absence of DR were selected. The demographic characteristics of the study population are shown in **Table 1**.

**Table 1:** Demographics, comorbidities and serum test results across groups.

Subjects, n	Control	T2DM	NPDR	PDR	P-value	P^a^	P^b^	P^c^	P^d^	P^e^	P^f^
	27	27	28	28							
Age Mean ± SD	67.18 ± 7.77	65.75 ± 7.64	68.72 ± 9.31	63.59 ± 6.97	0.107	0.913	0.891	0.348	0.516	0.749	0.083
Gender, n (%) Male	13 (46.4%)	11 (39.3%)	20 (69.0%)	16 (55.2%)	0.132						
BMI	23.86 ± 3.11	24.28 ± 3.65	24.88 ± 3.19	24.92 ± 2.92	0.563	0.963	0.646	0.614	0.903	0.883	1.000
Diabetes duration, y	0	10.54 ± 5.19	15.10 ± 7.95	23.83 ± 8.42	<0.001	<0.001	<0.001	<0.001	0.045	<0.001	<0.001
FPG (mm/L)	5.58 ± 0.71	7.40 ± 1.68	7.77 ± 2.09	8.69 ± 3.54	<0.001	0.021	0.003	<0.001	0.928	0.155	0.433
HbA1 (mm/L)	5.11 ± 0.59	6.99 ± 1.06	7.22 ± 1.04	7.46 ± 0.93	<0.001	<0.001	<0.001	<0.001	0.798	0.243	0.764
HDL-c (mm/L)	1.47 ± 0.36	1.28 ± 0.27	1.22 ± 0.26	1.20 ± 0.32	0.005	0.097	0.015	0.007	0.898	0.788	0.995
LDL-c (mm/L)	3.18 ± 0.86	3.00 ± 0.77	2.70 ± 1.17	2.79 ± 0.88	0.227	0.888	0.231	0.403	0.641	0.837	0.986
SCr (mm/L)	73.75 ± 17.50	70.1 ± 14.24	78.00 ± 19.70	95.14 ± 44.46	0.005	0.976	0.937	0.021	0.749	0.006	0.087
TG (mm/L)	1.55 ± 0.59	1.37 ± 0.58	1.83 ± 1.17	1.93 ± 1.49	0.172	0.916	0.743	0.532	0.347	0.192	0.986
BUN (mm/L)	5.04 ± 1.32	5.75 ± 1.46	6.08 ± 1.77	7.83 ± 3.11	<0.001	0.588	0.245	<0.001	0.932	0.002	0.010
HTN%	42.8%	42.9%	58.6.0%	65.5%	0.213						
Treatment OAD SII OAD + SII	— — —	14 5 8	10 6 12	6 7 15	0.226						

For age, diabetes duration, FPG, HbA1c, HDL-c, LDL-c, SCr, TG and BUN the mean and standard deviations are presented, and comparisons were made by Wilcoxon rank sum test. Gender, rates of comorbidities and treatment of diabetes were compared by X2 test. T2DM, type 2 diabetes mellitus; NPDR, non-proliferative diabetic retinopathy; PDR, proliferative diabetic retinopathy; BMI, body mass index; FPG, fasting plasma glucose; HbA1c, Hemoglobin A1c; HDL-c, High density lipoprotein- cholesterol; LDL-c, Low density lipoprotein- cholesterol; SCr, serum creatinine; TG, triglycerides; BUN, blood urea nitrogen; HTN, hypertension; OAD, oral antidiabetic drug; SII, subcutaneous insulin injection. P^a^, P-value of control subjects versus T2DM samples. P^b^, P-value of control subjects versus NPDR samples. P^c^, P-value of control subjects versus PDR samples. P^d^, P-value of T2DM samples versus NPDR samples. P^e^, P-value of T2DM samples versus PDR samples. P^f^, P-value of NPDR samples versus PDR samples.

### 3.2 Plasma metabolite differences between subjects grouped by different DR status

**Figure 2:**
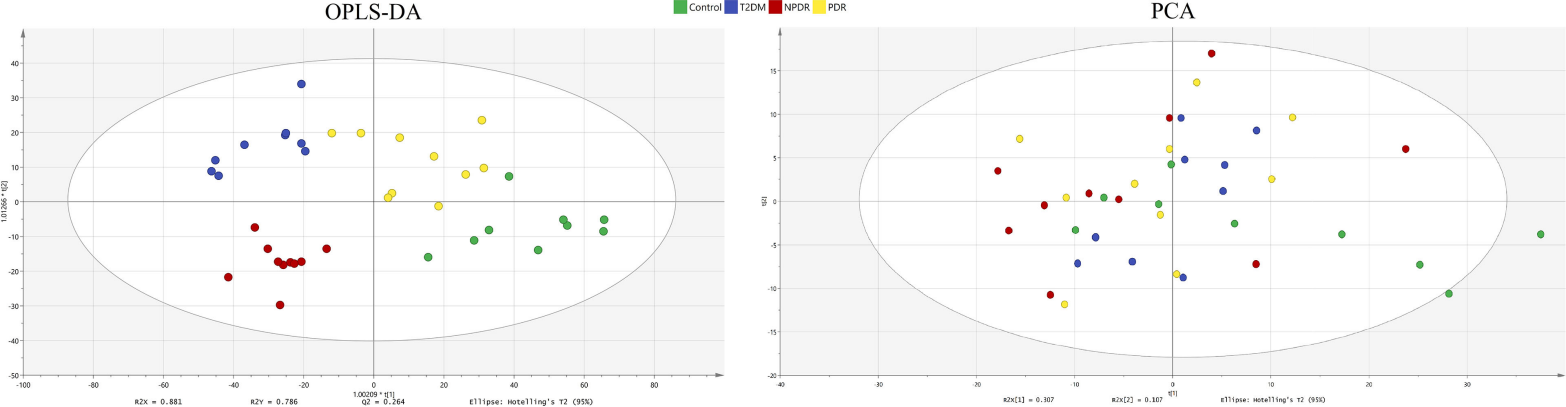
OPLS-DA model and PCA model. The four groups were well separated in the OPLS-DA score plot (R2X = 0.881, R2Y = 0.786 and Q2 = 0.264.) The PCA model for samples collected from 4 isolates of sample data. Green dot: control. Blue dot: T2DM sample. Red dot: NPDR sample. Yellow dot: PDR sample. OPLS-DA, orthogonal partial least squares- discriminant analysis; PCA, principal component analysis; T2DM, type 2 diabetes mellitus; NPDR, non-proliferative diabetic retinopathy; PDR, proliferative diabetic retinopathy.

**Figure 3:**
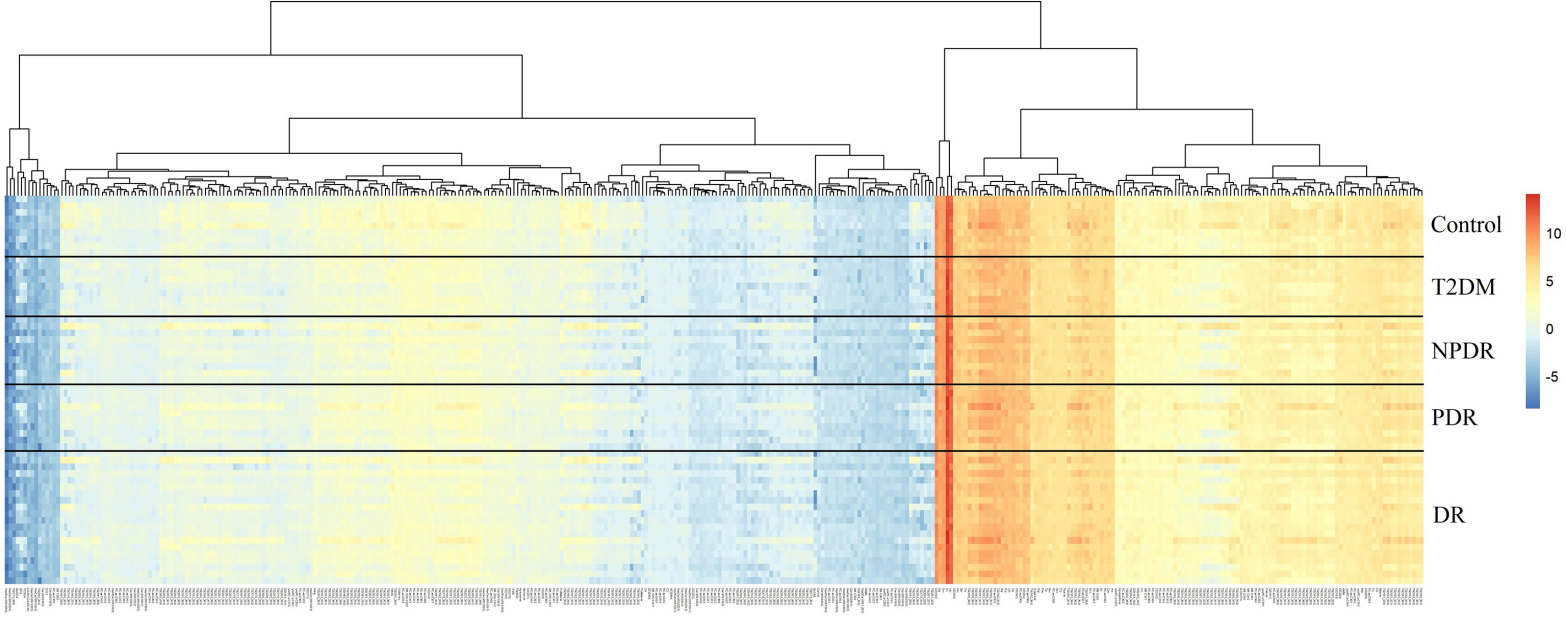
Cluster analysis showed that the identified metabolites were clearly grouped into controls, T2DM, NPDR and PDR sample clusters with high repeatability and the resulting data were reliable and logical. The distinctness of each group in the right and center could clearly be seen, and the blending of the groups were shown in the lefts. Significant metabolic features increased (red) or decreased (blue) compared with the others group. T2DM, type 2 diabetes mellitus; DR, diabetic retinopathy; NPDR, non-proliferative diabetic retinopathy; PDR, proliferative diabetic retinopathy.

**Figure 4:**
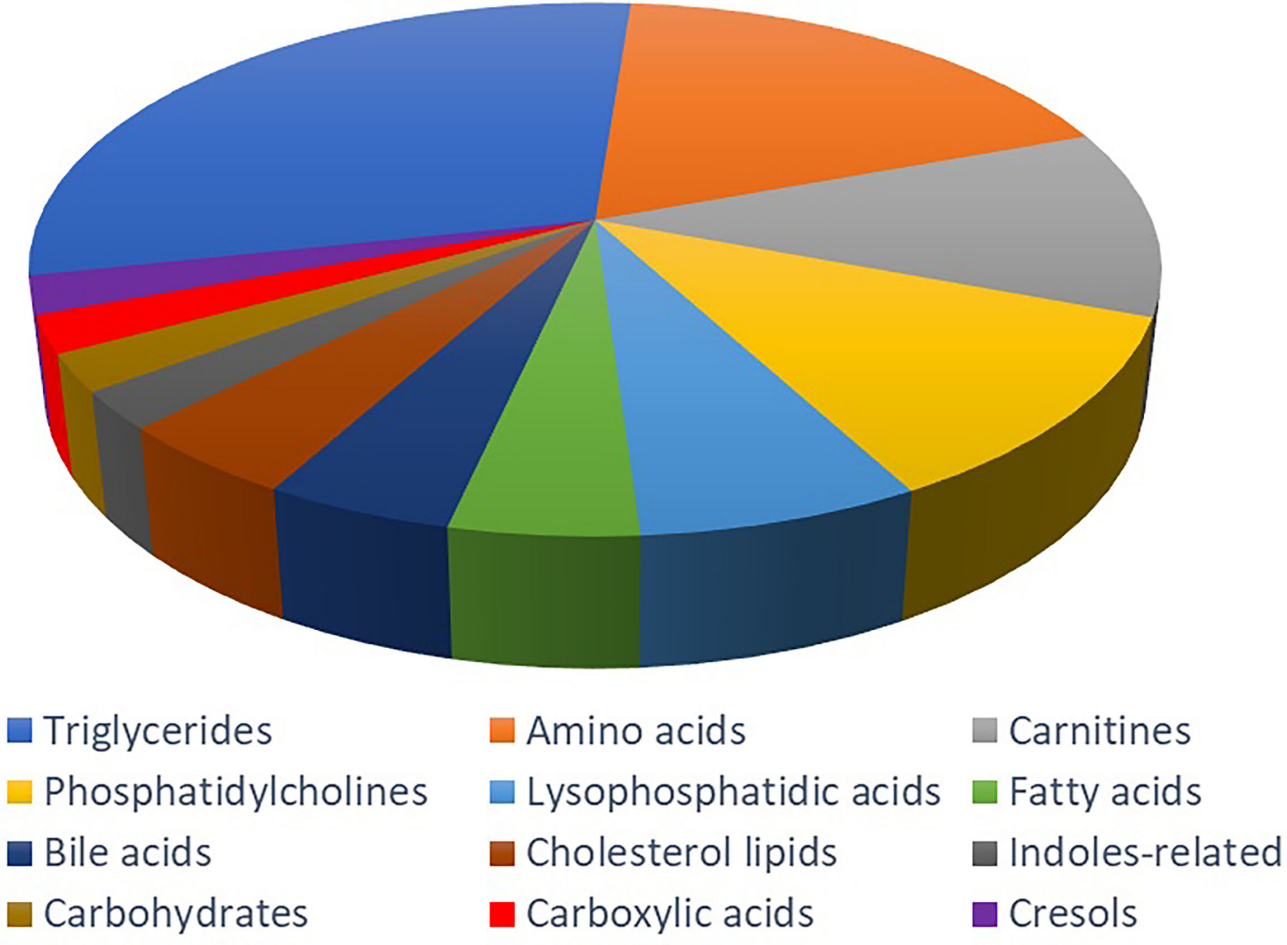
Metabolite classification analysis. The pie chart shows the 41 metabolites, including triglycerides (31.70%), amino acid (19.51%), carnitine (12.20%), phosphatidylcholine (12.20%), lysophosphatidic acid (7.32%), fatty acid (4.88%), bile acid (4.88%), cholesterol lipids (4.88%), indoles-related metabolites (2.44%), carbohydrate (2.44%), carboxylic acid (2.44%) and cresol (2.44%).

In the targeted metabolomics datasets, the OPLS-DA model with supervised methods in **Figure 2** showed that all four groups were clearly separated, which indicated the significant metabolic differences between each group. The principal component analysis (PCA) model for samples collected from the 4 isolates of sample data is shown in **Figure 2**. Clustering heatmap showed the relationship between the metabolite content clustering between groups. The identified metabolites in the controls, T2DM, NPDR and PDR groups showed distinguishable clusters in groups, even though the sample clusters overlapped slightly (**Figure 3**). UHPLC-MS/MS of targeted metabolomics was used to investigated and analyze 541 metabolites in the plasma samples of control subjects and different DR stages, of which 201 biomarkers significantly distinguished. According to the changes of these differential metabolites at different DR stages, 41 of these metabolites were considered as the potential markers to explain the key period variability in DR development. They were classified into 12 subcategories, of which glycerophospholipids had the highest percentage (31.7%) (**Figure 4**). To identify the metabolites responsible for these separations, variable importance in the projection (VIP), fold changes (FC) and p-value were mainly used. The VIP value is an important parameter for detecting potential biomarker candidates that reflects the correlation of the metabolites with different biological states. In our study, VIP values > 1.0 of OPLS–DAs were used. For evaluating statistical significance, p < 0.05 derived from t-test was applied. The relative metabolite levels were converted into FC which is the ratio of each metabolite to the mean of all biological repeat quantitative values between groups. FC > 1.2 and < 0.833 were used respectively to indicate the significantly up-regulated and down-regulated differential metabolites.

### 3.3 Potential biomarkers for targeted metabolomics in critical periods of DR development

#### 3.3.1 T2DM Versus NPDR

**Figure 5:**
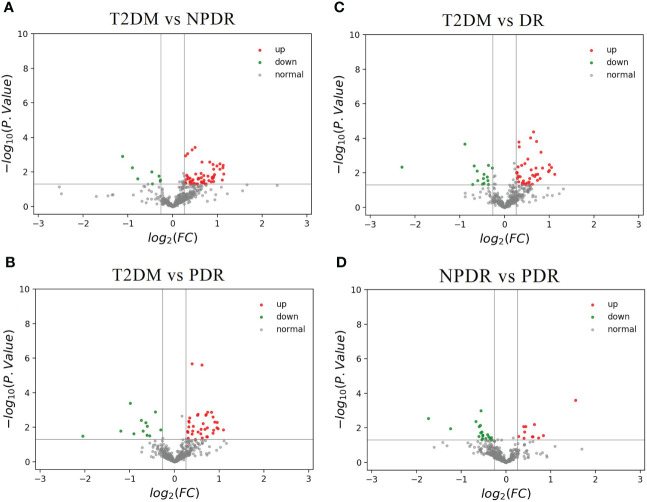
**(A)** The volcano map of the log2 (FC) and −log10 (p-value) showed that 65 differential metabolites were significantly different between T2DM samples (n = 9) and NPDR samples (n = 10). Compared with NPDR samples, 57 metabolic features were significantly increased (red dots) and 8 metabolic features were significantly decreased (green dots) in T2DM samples. **(B)** The volcano map of the log2 (FC) and −log10 (p-value) showed that 48 differential metabolites were significantly different between T2DM samples (n = 9) and PDR samples (n = 10). Compared with PDR samples, 36 metabolic features were significantly increased (red dots) and 12 metabolic features were significantly decreased (green dots) in T2DM samples. **(C)** The volcano map of the log2 (FC) and −log10 (p-value) showed that 56 differential metabolites were significantly different between T2DM samples (n = 9) and DR samples (n = 20). Compared with DR samples, 41 metabolic features were significantly increased (red dots) and 15 metabolic features were significantly decreased (green dots) in T2DM samples. **(D)** The volcano map of the log2 (FC) and −log10 (p-value) showed that 31 differential metabolites were significantly different between NPDR samples (n = 10) and PDR samples (n = 10). Compared with PDR samples, 12 metabolic features were significantly increased (red dots) and 19 metabolic features were significantly decreased (green dots) in NPDR samples. FC, fold change; T2DM, type 2 diabetes mellitus; NPDR, non-proliferative diabetic retinopathy; PDR, proliferative diabetic retinopathy.

Compared with T2DM and NPDR groups, 65 of the total 201 differential metabolites were detected, of which 57 biomarkers were higher in T2DM group, while the other 8 were lower (**Figure 5A**). Of the 41 metabolites we identified that distinguish critical period metabolites in DR development, 26 showed in this comparison group. Compared with T2DM group, the serum levels of alpha-aminobutyric acid (AABA), lactic acid, IAA, octadecanecarnitine and fatty acid 20:1 in NPDR group were higher, while the serum levels of Cit, taurocholic acid (TCA), carnitine, hexanoylcarnitine, cholesterol ester (CE) 16:1, 4 PCs (C32:1, C32:2, C36:6 and C42:4) and 12 triglycerides (TC) were lower.

#### 3.3.2 T2DM Versus PDR

The 48 of total 201 differential metabolites were found in T2DM versus PDR groups. Thirty-six of the differential metabolites were higher in T2DM group and the others were lower than PDR group (**Figure 5B**). Compared with PDR and T2DM groups, we found 28 differential metabolites in the critical periods of DR. The serum levels of beta-aminobutyric acid (BABA), 1-MH and phenylalanine betaine of amino acid, TCA of bile acid, p-cresol sulfate, acylcarnitine (C18:2) and fatty acid 20:1 in PDR group were higher than T2DM group. And the serum levels of CE 16:1, CE 22:5, 3 LPAs (C16:1, C26:0 and C28:1), butyrylcarnitine, 5 PCs (C32:1, C32:2, C34:4, C36:6 and C42:4) and 12 triglycerides in PDR group were lower.

#### 3.3.3 T2DM Versus DR (including NPDR and PDR)

In the comparison of T2DM and DR groups, 56 of the 201 differential metabolites were detected, of which the serum levels of 41 biomarkers in DR group were higher in T2DM group, while the serum levels of the other 15 biomarkers were lower in T2DM group (**Figure 5C**). Furthermore, in the 41 discriminating metabolites we identified contributed to the critical periods of DR development, as 31 of which could be found in this comparison group. Compared with T2DM group, the serum levels of 5 amino acid-related metabolites (BABA, 1-MH, 3-methylhistidine (3-MH), AABA and phenylalanine betaine), lactic acid, IAA, acylcarnitine (C18:2), and fatty acid 20:1 were higher, and the levels of TCA, carnitine, hexanoylcarnitine, butyrylcarnitine, CE 16:1, 5 PCs (C32:1, C32:2, C34:4, C36:6 and C42:4) and 13 triglycerides were lower in DR group.

#### 3.3.4 NPDR Versus PDR

Through targeted metabolomics, a total of 31 of the 201 differential metabolite were found in the comparison of NPDR and PDR, of which serum levels of 12 key metabolites were higher in NPDR group, while 19 were lower (**Figure 5D**). And 10 of 41 critical metabolites were further detected. Serum levels of 5-aminovaleric acid and p-cresol sulfate in PDR group were higher than that of NPDR group, while serum levels of alanine, CDCA, IAA, butyrylcarnitine, EPA, CE 22:5 and 2 PCs (C34:4 and C36:6) in PDR group were lower than that of NPDR group.

### 3.4 Intercomparison and validation of the result of targeted and untargeted metabolomics

**Figure 6:**
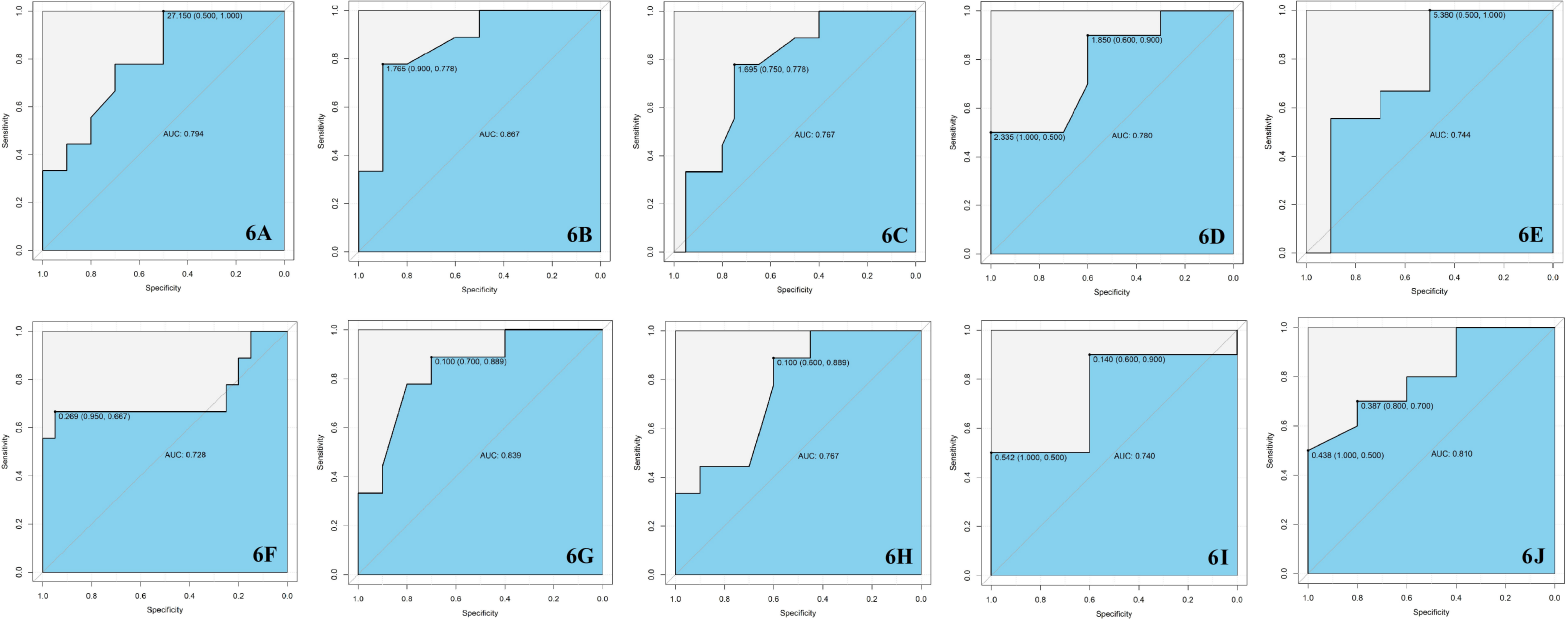
**(A)** The serum level of Cit in T2DM group was higher than NPDR group with the AUC = 0.794. **(B)** The serum level of IAA in T2DM group was lower than NPDR group with the AUC = 0.867. **(C)** The serum level of IAA in T2DM group was lower than DR group with the AUC = 0.767. **(D)** The serum level of IAA in NPDR group was higher than PDR group with the AUC = 0.780. **(E)** The serum level of 1-MH in T2DM group was lower than PDR group with the AUC = 0.744. **(F)** The serum level of 1-MH in T2DM group was lower than DR group with the AUC = 0.728. **(G)** The serum level of hexanoylcarnitine in T2DM group was higher than NPDR group with the AUC = 0.839. **(H)** The serum level of hexanoylcarnitine in T2DM group was higher than DR group with the AUC = 0.767. **(I)** The serum level of CDCA in NPDR group was higher than PDR group with the AUC = 0.740. **(J)** The serum level of EPA in NPDR group was higher than PDR group with the AUC = 0.810. AUC, area under the curve; T2DM, type 2 diabetes mellitus; DR, diabetic retinopathy; NPDR, non-proliferative diabetic retinopathy; PDR, proliferative diabetic retinopathy; Cit, L-Citrulline; IAA, Indoleacetic acid; 1-MH, 1-Methylhistidine; CDCA, Chenodeoxycholic acid; EPA, Eicosapentaenoic acid.

After searching as many differential metabolites as possible through targeted metabolomics, we compared the results with our previous untargeted metabolomics results and found that a total of 7 biomarkers in the critical period of DR, including Cit, IAA, 1-MH, PCs, hexanoylcarnitine, CDCA and EPA were detected in both targeted and untargeted metabolomic analyses (**Table 2**). In targeted metabolomics, we found that the serum level of Cit in NPDR group were lower than those in the T2DM group (AUC = 0.794, **Figure 6A**), whereas our previous untargeted metabolomic analysis showed that the serum Cit level in DR group were higher than in T2DM group. In terms of serum IAA, we found that serum IAA levels in NPDR group and DR group were significantly higher than those in T2DM group (AUC = 0.867, **Figure 6B** and AUC = 0.767, **Figure 6C**) through targeted metabolomics. Our previous untargeted metabolomics studies also observed the higher IAA level in DR group than in T2DM group. In addition, we further found that the serum level of IAA was significantly lower in PDR group than in NPDR group (AUC = 0.780, **Figure 6D**) in the targeted metabolomics, which has not been reported. In our study, we found that serum level of 1-MH in PDR group were significantly higher than those in T2DM group in both targeted metabolomic (AUC = 0.744, **Figure 6E**) and untargeted metabolomic analyses, and were also significantly higher in DR group compared with T2DM group in targeted metabolomic analysis (AUC = 0.728, **Figure 6F**). In our previously diabetic retinopathy-untargeted metabolomics, the level of PC C16:0 in serum was significantly positively correlated with the severity of DR. Conversely, in targeted metabolomic analyses, the serum of PCs (including PC C32:1, C32:2, 34:4, C36:6 and C42:2) were inversely proportional to the degree of progression of DR. In terms of serum carnitine levels, both carnitine and hexyl carnitine (AUC = 0.839, **Figure 6G**) in the NPDR group were lower than those in the T2DM group in our targeted and untargeted metabolomics analysis. These results were the same as the comparison between the DR group and the T2DM group (AUC = 0.767, **Figure 6H**). Besides, the serum level of butylcarnitine in PDR group was significantly lower than that in NPDR group and T2DM group in targeted metabolomics, while according to our previously untargeted metabolomic analysis, the serum levels of caproylcarnitine and palmitoylcarnitine in PDR group were significantly lower than those in T2DM group, and the serum palmitylcarnitine level was even lower than that in NPDR group. Furthermore, in our previous studies, the serum level of CDCA in NPDR group and DR group were significantly higher than that in T2DM group by untargeted metabolomics, but in targeted metabolomics, we found that the level of serum CDCA in PDR group was lower compared with NPDR group (AUC = 0.740, **Figure 6I**). In addition, we also found that the level of serum UDCA in PDR group and DR group were significantly lower than T2DM group through untargeted metabolomics, and the serum level of TCA in PDR group, NPDR group and DR group were all significantly lower than T2DM group through targeted metabolomics. However, UDCA and TCA have not been found in both targeted and untargeted metabolomics so far. Regarding targeted and untargeted metabolomics, we found that the serum EPA level in PDR group was significantly lower than that in NPDR group (AUC = 0.810, **Figure 6J**). In addition, in untargeted metabolomics, serum DHA levels in PDR group were significantly lower than those in NPDR group and T2DM group, respectively.

**Table 2:** Metabolites of DR critical period identified from targeted and untargeted metabolomic profiling (**13**).

	Targeted Metabolomics				Untargeted Metabolomics			
	T2DM vs. NPDR	T2DM vs. PDR	T2DM vs. DR	NPDR vs. PDR	T2DM vs. NPDR	T2DM vs. PDR	T2DM vs. DR	NPDR vs. PDR
L-Citrulline Ratio P-value	1.501 0.008	**—**	**—**	**—**	**—**	**—**	0.619 0.036	—
Indoleacetic acid Ratio P-value	0.536 0.001	—	0.653 0.009	1.516 0.007	—	—	0.681 0.013	—
1-Methylhistidine Ratio P-value	—	0.683 0.031	0.771 0.028	—	—	0.486 0.031	—	—
Hexanoylcarnitine Ratio P-value	1.396 0.044	—	1.385 0.043	—	—	1.594 0.008	—	—
Chenodeoxycholic acid Ratio P-value	—	—	—	9.819 0.011	0.344 0.011	—	0.316 < 0.001	—
Eicosapentaenoic acid Ratio P-value	—	—	—	1.784 0.010	—	1.667 < 0.001	—	1.948 0.012
Phosphatidylcholines PC C16:0 Ratio P-value	—	—	—	—	—	0.680 < 0.001	0.753 0.003	0.805 0.014
PC C32:1 Ratio P-value	1.427 0.012	1.440 0.002	1.434 0.002	—	—	—	—	—
PC C32:2 Ratio P-value	1.366 0.027	1.632 0.002	1.494 < 0.001	—	—	—	—	—
PC C34:4 Ratio P-value	—	1.862 0.005	1.592 0.016	1.321 0.039	—	—	—	—
PC C36:6 Ratio P-value	1.309 0.041	1.773 0.001	1.506 0.007	1.354 0.009	—	—	—	—
PC C42:4 Ratio P-value	1.260 0.001	1.252 0.005	1.256 <0.001	—	—	—	—	—

PC, Phosphatidylcholine; T2DM, type 2 diabetes mellitus; NPDR, non-proliferative diabetic retinopathy; PDR, proliferative diabetic retinopathy; DR, diabetic retinopathy.

### 3.5 Revalidate the differential metabolites by ELISA

As the ELISA kits for the detection of 1-MH, hexanoylcarnitine and PC are unavailable commercially, and metabolomics is considered to be the best method for detecting small molecule metabolites such as carnitine and fatty acids currently, we only re-validated the other 4 differential metabolites, including Cit, IAA, CDCA and EPA.

We performed ELISA test on the serum of 18 T2DM samples, 18 PDR samples, 18 PDR samples and 18 controls (**Table 3**). We found that the serum Cit levels in controls, T2DM, NPDR, PDR and DR (NPDR and PDR) groups were 286.68 ± 85.17 pg/ml, 500.11 ± 276.85 pg/ml, 180.52 ± 110.30 pg/ml, 169.37 ± 141.23 pg/ml and 174.94 ± 126.83 pg/ml, respectively. Compared with controls, NPDR, PDR and DR groups, the serum level of Cit in T2DM group was significantly higher (P = 0.001, < 0.001, < 0.001 and < 0.001). This result was similar to the targeted metabolomics which indicated the serum level of Cit in T2DM group was higher than NPDR group. The serum levels of IAA were 70.47 ± 23.80 ng/ml, 53.33 ± 16.66 ng/ml, 83.48 ± 20.29 ng/ml, 93.16 ± 37.28 ng/ml and 88.32 ± 30.40 ng/ml in controls, T2DM, NPDR, PDR and DR (NPDR and PDR) groups respectively. Compared with T2DM, we found that the serum levels of IAA in NPDR, PDR and DR group were significantly higher (P = 0.014, < 0.001 and < 0.001), which conformed to the results of our targeted and untargeted metabolomics results. The IAA serum level in NPDR group was higher than T2DM group from targeted metabolomics, and the IAA serum levels in DR group were higher than T2DM group from both targeted and untargeted metabolomics. However, although we found higher serum levels of IAA in NPDR group than in PDR group in targeted metabolomics, this was not detected in the ELISA test. Through ELISA test, the serum levels of CDCA in controls, T2DM, NPDR, PDR and DR (NPDR and PDR) groups were 1651.27 ± 577.20 nmol/L, 2650.36 ± 469.08 nmol/L, 2022.46 ± 710.91 nmol/L, 826.51 ± 667.37 nmol/L and 1426.30 ± 888.79 nmol/L. The serum level of CDCA in T2DM group was significantly higher than those in controls, PDR and DR groups, respectively (P = 0.001, <0.001 and <0.001). Compared with PDR group, the serum level of CDCA was also higher in controls and NPDR group (P = 0.013 and < 0.001) which was consistent with our targeted metabolomics results. The results of ELISA test in EPA showed that the serum EPA levels in controls, T2DM, NPDR, PDR and DR (NPDR and PDR) groups were 312.45 ± 47.91 pg/ml, 263.19 ± 38.20 pg/ml, 256.05 ± 27.69 pg/ml, 196.51 ± 22.55 pg/ml and 226.28 ± 39.03 pg/ml, respectively. Serum EPA levels in the control group were significantly higher than those in the others groups (P = 0.001 in control vs. T2DM, P < 0.001 in control vs. NPDR, P < 0.001 in control vs. PDR and P < 0.001 in control vs. DR). And compared with PDR and DR groups, the serum level of EPA was higher in T2DM group (P < 0.001 and 0.008). Of note, the level of EPA in serum was also higher in NPDR group than in PDR group (P < 0.001), which was fully consistent with the results from both targeted and untargeted metabolomics.

**Table 3:** Validate the Differential Metabolites in control, T2DM, NPDR, PDR and DR groups by ELISA.

	L-Citrulline(pg/ml)	Indoleacetic Acid(ng/ml)	Chenodeoxycholic Acid (nmol/L)	Eicosapentaenoic Acid (pg/ml)
Subjects, n	18	18	18	18
Control	286.68 ± 85.17	70.47 ± 23.80	1651.27 ± 577.20	312.45 ± 47.91
T2DM	500.11 ± 276.85	53.33 ± 16.66	2650.36 ± 469.08	263.19 ± 38.20
NPDR	180.52 ± 110.30	83.48 ± 20.29	2022.46 ± 710.91	256.05 ± 27.69
PDR	169.37 ± 141.23	93.16 ± 37.28	826.51 ± 667.37	196.51 ± 22.55
DR	174.94 ± 126.83	88.32 ± 30.40	1426.30 ± 888.79	226.28 ± 39.03
P-value	< 0.001	< 0.001	< 0.001	< 0.001
P^a^	0.001	0.359	0.001	0.001
P^b^	0.284	0.633	0.570	< 0.001
P^c^	0.193	0.116	0.013	< 0.001
P^d^	0.122	0.185	0.830	< 0.001
P^e^	< 0.001	0.014	0.093	0.979
P^f^	< 0.001	< 0.001	< 0.001	0.008
P^g^	< 0.001	< 0.001	< 0.001	< 0.001
P^h^	> 0.999	0.838	< 0.001	< 0.001
P^i^	> 0.999	0.975	0.050	0.054
P^j^	> 0.999	0.975	0.061	0.054

T2DM, type 2 diabetes mellitus; NPDR, non-proliferative diabetic retinopathy; PDR, proliferative diabetic retinopathy; DR, diabetic retinopathy. P^a^, P-value of control subjects versus T2DM samples. P^b^, P-value of control subjects versus NPDR samples. P^c^, P-value of control subjects versus PDR samples. P^d^, P-value of control subjects versus DR samples. P^e^, P-value of T2DM samples versus NPDR samples. P^f^, P-value of T2DM samples versus PDR samples. P^g^, P-value of T2DM samples versus DR samples. P^h^, P-value of NPDR samples versus PDR samples. P^i^, P-value of NPDR samples versus DR samples. P^j^, P-value of PDR samples versus DR samples.

### 3.6 Analysis the differential metabolites between groups by multiple linear regression

**Figure 7:**
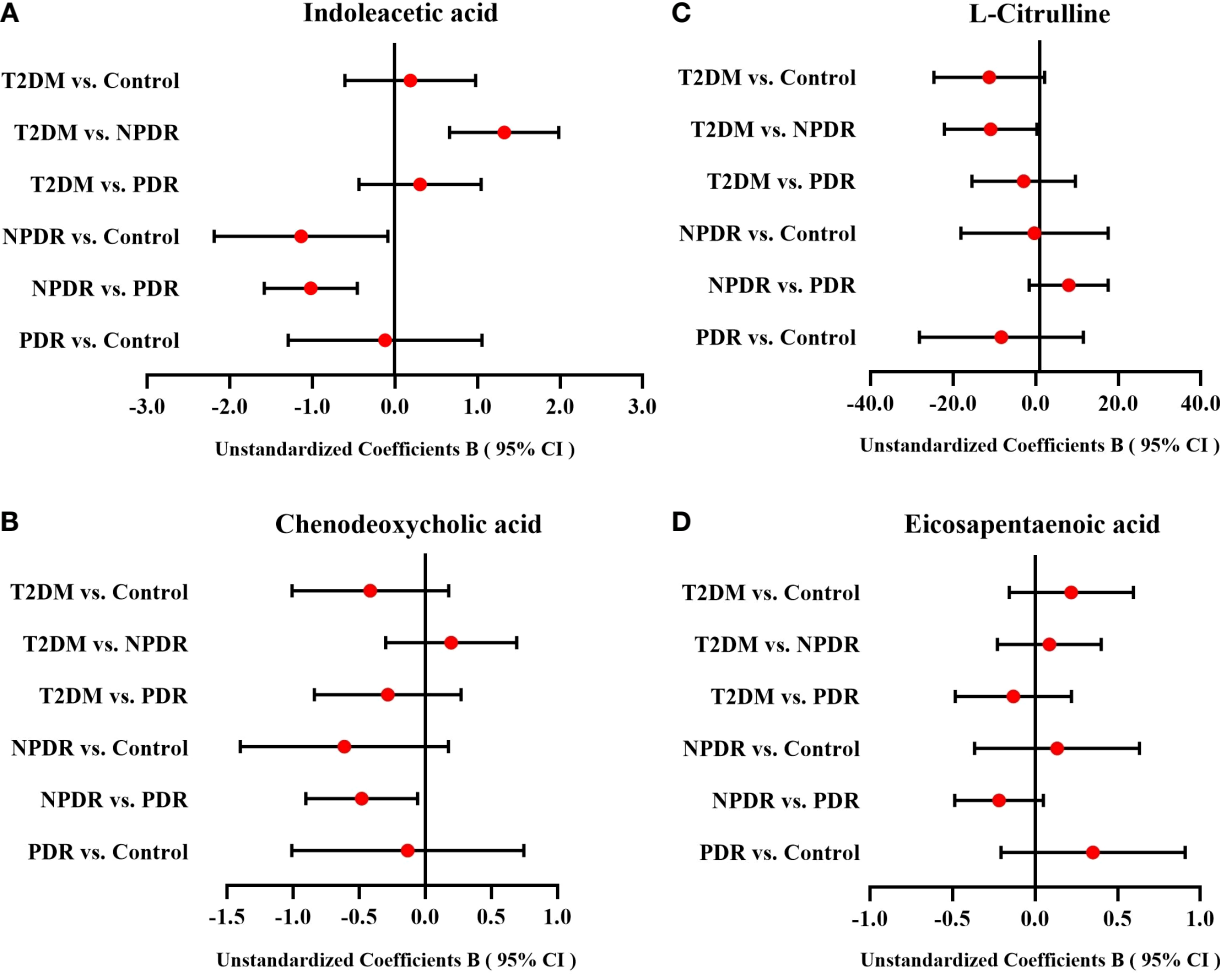
The forest plots of IAA, CDCA, Cit and EPA among groups. After multiple linear regression analyses of age, diabetes duration, FPG and HbA1 for differential metabolites between groups, **(A)** the serum level of IAA between T2DM group and NPDR group, NPDR group and control group, and NPDR group and PDR group were statistically significant, **(B)** the serum level of CDCA between NPDR group and PDR group was also statistically significant, and **(C)** the serum Cit level between T2DM group and NPDR group was of borderline statistical significance. **(D)** The serum levels of EPA which showed no statistically significant difference among the groups. CI, confidence interval; T2DM, type 2 diabetes mellitus; NPDR, non-proliferative diabetic retinopathy; PDR, proliferative diabetic retinopathy; Cit, L-Citrulline; IAA, Indoleacetic acid; 1-MH, 1-Methylhistidine; CDCA, Chenodeoxycholic acid; EPA, Eicosapentaenoic acid.

After comparison of targeted and untargeted metabolomics results, and cross-validation by Elisa, we mainly indicated that the DR stage showed lower serum level of Cit and higher serum level of IAA compared with the T2DM stage, and the serum levels of CDCA and EPA in PDR stage were significantly lower than NPDR stage. However, since age, diabetes duration, FPG, and HbA1 based on **Table 1** may influence the significance of differential metabolites between groups, we performed multiple linear regression analysis. We found that after adjusting age, diabetes duration, FPG and HbA1 of patients in each group, the serum levels of IAA were statistically significant in NPDR vs Control (P = 0.035), T2DM vs NPDR (P < 0.001) and NPDR vs PDR (P = 0.001), the serum CDCA level was statistically significant in NPDR vs PDR (P = 0.028), and the serum Cit level was also of borderline statistical significance in T2DM vs NPDR (P = 0.056). However, the serum levels of EPA showed no statistically significant difference among the groups. (**Table 4** and **Figure 7**).

**Table 4:** The multiple linear regression result of the differential metabolites between groups.

	Indoleacetic acid		Chenodeoxycholic acid		L-Citrulline		Eicosapentaenoic acid	
Groups	Unstandardized Coefficients B (95.0% CI)	P-value	Unstandardized Coefficients B (95.0% CI)	P-value	Unstandardized Coefficients B (95.0% CI)	P-value	Unstandardized Coefficients B (95.0% CI)	P-value
Age	-0.021 (-0.055, 0.014)	0.229	0.006 (-0.020, 0.032)	0.635	0.034 (-0.546, 0.614)	0.905	0.013 (-0.003, 0.030)	0.105
Diabetes duration	0.013 (-0.023, 0.048)	0.472	-0.008 (-0.035, 0.018)	0.535	-0.450 (-1.050, 0.150)	0.136	0.000 (-0.017, 0.016)	0.967
FPG	0.057 (-0.028, 0.143)	0.181	0.042 (-0.022, 0.106)	0.193	1.019 (-0.432, 2.470)	0.162	-0.003 (-0.044, 0.038)	0.881
HbA1	-0.176 (-0.423, 0.070)	0.154	-0.102 (-0.287, 0.082)	0.266	-1.907 (-6.083, 2.269)	0.358	0.019 (-0.098, 0.136)	0.746
T2DM vs. Control	0.187 (-0.604, 0.978)	0.632	-0.415 (-1.007, 0.178)	0.163	-11.207 (-24.613, 2.199)	0.098	0.220 (-0.156, 0.596)	0.241
T2DM vs. NPDR	1.323 (0.663, 1.984)	< 0.001	0.197 (-0.298, 0.692)	0.423	-10.923 (-22.122, 0.276)	0.056	0.087 (-0.227, 0.401)	0.577
T2DM vs. PDR	0.305 (-0.436, 1.046)	0.407	-0.283 (-0.838, 0.272)	0.306	-2.905 (-15.463, 9.654)	0.640	-0.131 (-0.483, 0.221)	0.452
NPDR vs. Control	-1.136 (-2.188, -0.084)	0.035	-0.612 (-1.399, 0.176)	0.123	-0.284 (-18.108, 17.541)	0.974	0.134 (-0.366, 0.633)	0.589
NPDR vs. PDR	-1.019 (-1.583, -0.454)	0.001	-0.480 (-0.903, -0.057)	0.028	8.018 (-1.555, 17.592)	0.097	-0.218 (-0.486, 0.050)	0.108
PDR vs. Control	-0.118 (-1.290, 1.055)	0.839	-0.132 (-1.010, 0.746)	0.761	-8.302 (-28.177, 11.573)	0.400	0.351 (-0.206, 0.909)	0.207

T2DM, type 2 diabetes mellitus; NPDR, non-proliferative diabetic retinopathy; PDR, proliferative diabetic retinopathy; FPG, fasting plasma glucose; HbA1c, Hemoglobin A1c; CI, confidence interval.

## 4 Discussion

Metabolomics as a powerful approach for studying pathophysiological processes can be divided into untargeted metabolomics and targeted metabolomics. Untargeted metabolomics reflects the multivariate dynamic changes of all metabolite levels as much as possible which is helpful to identify unknown disease mechanisms, while targeted metabolomics more accurately detects and analyzes specific metabolites in biological samples. Since the results of metabolomics tests are influenced by the selection of different test methods and sample characteristics (e.g., race, gender, age, dietary structure, environment, and drugs), the results of studies in different regions have a certain degree of difference. At present, there have been many metabolomics studies on diabetic retinopathy, among which the serum (1, 18–21), vitreous and aqueous humor (22–25) of samples are mainly used as samples for metabolomics detection. However, since sampling of the vitreous and the aqueous humor are invasive and their repeatability of detection are difficult, greatly limit their value in studying the metabolomics of DR. In contrast, serum remains the best sample choice for metabolomic testing. To our knowledge, we are the first double comparison study of untargeted metabolomics and targeted metabolomics by LC-MS in Chinese with different severities of DR and using ELISA to further cross-validate the key metabolites.

We found that in both targeted and untargeted metabolomic assays, Cit, IAA, 1-MH, PCs, hexanoylcarnitine, CDCA and EPA were detected and showed significantly different between groups of samples with different degrees of DR. After further analysis, we mainly concluded that samples in the DR stage showed lower serum level of Cit and higher serum level of IAA compared with samples in the T2DM stage, while during the progression of diabetic retinopathy, the serum levels of CDCA and EPA in PDR stage were significantly lower than NPDR stage. Although these biomarkers were regarded as differential metabolites in both targeted and untargeted metabolomics, there were still differences in their expression levels between groups.

Under normal circumstances, L-arginine and Cit can be converted into each other through various pathways in humans. L-arginine is metabolized by nitric oxide synthase (NOS) to produce nitric oxide and Cit. Cit can be recycled back to L-arginine by argininosuccinate synthase and argininosuccinate lyase (26). Due to the dysregulation of nitrogen metabolites-related pathways in DR samples, particularly maladjusted arginine and citrulline, the serum Cit level in diabetic samples is disordered, which leads to the dysfunction of retinal endothelial cell (1). The result of untargeted metabolomics analysis in our study indicated that the serum level of Cit was higher in DR group compared with T2DM group, which was consistent with the results of the serum non-targeted metabolomics of DR by Sumarriva K et al. (1) and also similar to a vitreous untargeted metabolomics of DR in 2018 (27). However, we found that in targeted metabolomics, NPDR group had lower serum Cit level than T2DM group (AUC = 0.794, **Figure 6A**), which also have been reported in the global amino acid profile of DR status (28). In addition, a targeted metabolomics report in 2021 also showed that serum Cit level was lower in samples with impaired fasting glucose (IFG) compared to normal individuals (18). Therefore, we speculated that the difference in serum Cit levels between targeted and untargeted metabolomics may be due to the choice of different metabolomics methods and the comparison between different DR stages in each study.

Tryptophan is the main precursor of IAA synthesis, which is similar in chemical structure to IAA, and its degradation products include indoxyl sulfate and indoleacetic acid (29) (30). According to KEGG global metabolic network, tryptophan metabolism is one of the most disturbed metabolic pathways. Several studies have already demonstrated dysregulation of serum tryptophan level in DR samples (28) (18, 20, 31). However, the changes of IAA in serum level of DR have been rarely reported. Kong et al. suggested that increasing the levels of tryptophan and IAA and decreasing the level of indole acetaldehyde by drugs may modulate tryptophan metabolism to protect the nervous system of T2DM samples (32). Besides, there was a human trial showed that oral IAA can reduce blood glucose in diabetic samples (33). In 2022, Guo et al. observed that compared with T2DM samples, the serum level of IAA in DR samples was significantly higher (34), which was consistent with our results of targeted and untargeted metabolomics. Through targeted metabolomics, we found that serum IAA levels in both NPDR and DR groups were significantly higher than those in T2DM group (AUC = 0.867, **Figure 6B** and AUC = 0.767, **Figure 6C**), and our previous untargeted metabolomics studies also observed higher serum IAA level in DR group than T2DM group. Notably, we further found that the serum level of IAA in PDR group was significantly lower than NPDR group in targeted metabolomics (AUC = 0.780, **Figure 6D**), which has not been reported before.

Bile acids (BAs) are cholesterol catabolites that are mainly synthesized in the liver (35). In alternative pathways of BA synthesis, CDCA and cholic acid (CA) as two primary BA are formed predominantly in the pericentral hepatocytes over several steps from cholesterol (36) (37). Studies have shown that BAs can be involved in glucose metabolism and energy regulation. Some of the level of serum BAs are also affected by drugs and other biochemical indicators. In 2021 a cross-sectional study comparing serum bile acid levels in T2DM samples and non-T2DM samples, Mantovani et al. concluded that the level of serum CDCA in T2DM samples was not affected by statin, metformin, or incretins, and was significantly different from nondiabetic control individuals and T2DM samples with no drug therapy. In addition, level of serum TCA was lower in T2DM samples treated with incretins, and was significantly correlated with fasting glucose levels, while serum triglycerides were only significantly correlated with UDCA (38). UDCA was considered to have neuroprotective effects in retinal diseases (39) (40), and its inhibitory activity against to VEGF-induced pro-angiogenic and pro-permeabilization of human retinal microvascular endothelial cells was confirmed in the oxygen-induced retinopathy (OIR) mouse models (41). The conclusions of above studies are similar to our findings. In untargeted metabolomics, the serum level of CDCA in NPDR group and DR group was significantly higher than that in T2DM group, but in targeted metabolomics, we found that the level of serum CDCA in PDR group was lower compared with NPDR group (AUC = 0.740, **Figure 6I**). In addition, through untargeted metabolomics, we also found that the level of serum UDCA in PDR group and DR group was significantly lower than T2DM group, and the serum level of TCA in PDR group, NPDR group and DR group were significantly lower than T2DM group through targeted metabolomics. These results were similar to the previous studies on the relationship between T2DM and BAs.

Omega-3 long-chain polyunsaturated fatty acids (n-3 LC-PUFAs) as essential fatty acids in the human diet mainly including EPA and DHA which are expressed at high levels in the retina (42, 43). They have the function of regulating many biological processes, such as regulating vascular endothelial growth factor (VEGF) expression, preventing pericyte loss from retinal vascular inflammation, maintaining retinal capillary structure and integrity, and inhibiting retinal neovascularization (44–46). Numerous studies have found that n-3 LC-PUFAs are reduced in diabetic samples’ retina and serum, and researchers believed that increasing the intake of n-3 LC-PUFAs could help reduce the occurrence and development of DR (Saenz 47–50), which has been proved in diabetic animal models (45, 51). Our DR metabolomics study also confirmed the above statement. The serum level of EPA in the PDR group was significantly lower than NPDR group in both targeted (AUC = 0.810, **Figure 6J**) and untargeted metabolomic analysis. Besides, in the untargeted metabolomics, we also observed that the serum level of DHA in the PDR group was significantly lower compared with the NPDR group and the T2DM group, respectively. We hypothesize that DR could lead to damage of retinal vascular endothelial cells, excessive production of intracellular reactive oxygen species (ROS) and imbalance of VEGF expression, thus affecting the changes of n-3 LC-PUFAs levels in serum. However, a 2018 metabolomic study of NPDR samples found there was no difference in the serum levels of DHA and EPA in the NPDR group compared with health control (52). The other study on the relationship between diabetic retinopathy and lipid metabolism suggested that n-6 PUFAs (including linoleic acid, γ-linolenic acid, eicosadienoic acid, dihomo-γlinolenic acid and arachidonicacid) may be the potential indicators in distinguishing DR from other T2DM samples (53).

The results of our targeted metabolomics were basically consistent with those of ELISA in Cit, IAA, CDCA and EPA, while the results of untargeted metabolomics were only partially the same as those of ELISA in IAA and EPA. We believed that this may be due to the difference in metabolomics detection methods and the different thresholds of metabolites that can be detected by different metabolomics. Untargeted metabolomics is the identification of metabolites by comparing the obtained data with the standard product database after quantitative analysis. Targeted metabolomics, on the other hand, is to identify the specific target metabolite more precisely through kits of known metabolites. Therefore, through this comparative study of targeted and untargeted metabolomics, we believe that the accuracy of targeted metabolomics for the expression of the metabolites in serum is higher than that of untargeted metabolomics to a certain extent. To sum up, since the results of targeted and untargeted metabolomics were not completely consistent, in order to have a more comprehensive understanding of the occurrence and development of DR samples, the results of both methods should be evaluated at the same time, and the analysis and judgment should be made based on the sample’s current DR stage and the levels of differential metabolites in the sample’s serum.

In this study, after comparing targeted and untargeted metabolomics, we found that some of the major differential metabolites seemed not to appear in only one comparison group. Therefore, by performing the cross-validation of differential metabolites of Elisa, we further concluded that the serum level of Cit might be one of the main differential metabolites between T2DM stage and DR stage, and the serum level of CDCA might be a key biomarker which was significant different between NPDR stage and PDR stage. However, IAA and EPA need further discussions to clarify their meanings in different DR stages. In terms of IAA, through the double verification of metabolomics and Elisa, we found that the serum levels of IAA in both DR and NPDR groups were significantly higher than that in the T2DM group. However, since the DR group is composed of the NPDR group and PDR group, and the natural course of DR is mostly from the T2DM stage without DR to the DR stage including NPDR and PDR, we can reasonably infer that IAA may be the main differential metabolite that mainly appears in the progression of T2DM stage to DR stage. In terms of EPA, similarly, through metabolomics and Elisa, we found that compared with the PDR group, the serum IAA levels were significantly higher in both NPDR group and T2DM group, but there was no statistical difference between the NPDR group and T2DM group. As mentioned above, for most of the T2DM patients, the regular process of the DR progression is from the manifestation of non-DR to NPDR, and finally to PDR. Therefore, in contrast, we ultimately indicated that during the progression of DR, the change in the serum level of EPA from NPDR period to PDR period was more markedly different. In summary, we speculated that the serum levels of Cit and IAA might be the main differential metabolites between the periods of T2DM and DR, while the serum levels of CDCA and EPA might be the key biomarkers between the NPDR and PDR stages.

Several advantages can be found in the current study compared with previous studies. First, we used the widely targeted metabolomics approach to detect serum metabolites at different stages of DR samples, compared to our previous high resolution untargeted metabolomic results, and re-validated the differences of these biomarkers in different critical periods of DR. Compared with the traditional studies that only used untargeted metabolomic or targeted metabolomic analyses, our study seems be more comprehensive and accurate in comparing targeted and untargeted metabolomics and obtaining predefined metabolites. Secondly, participants in this study were recruited from the same region and were matched for age and gender to avoid potential confounding factors, making it more comparable between DR groups and controls. Thirdly, different from the grouping method of previous diabetic retinopathy metabolomics studies, we divided the participants into 4 groups, including the controls, T2DM, NPDR and PDR, and further analyzed the differences between the DR group and T2DM group. To our knowledge, this study is the first to confirm that IAA, 1-MH and CDCA are closely related to the progression of DR in humans. The changes in serum levels of Cit, PC, caproylcarnitine and EPA in our findings were not completely the same to those in previous studies. Thus, more rigorous and well-designed studies are needed to validate our findings. In addition, we further adopted multiple linear regression to analyze the differential metabolites between groups. After adjusting age, diabetes duration, FPG and HbA1, we found that the serum level of IAA, CDCA and Cit were still statistically significant in certain groups which were consistent with our results of metabolomics analysis and Elisa. However, the serum level of EPA was not statistically significant different among the groups. We speculated that this may be due to the small sample size of the study which may affect the reliability of the results to some extent. We are recruiting more participants based on the results of this study, and we plan to proceed with a larger clinical trial to obtain more meaningful and accurate results and further validate our results. In conclusion, our findings may provide some new clues and ideas for research on the prevention and development of DR, have the opportunity to better identify early NPDR samples in T2DM samples, and help distinguish NPDR samples from PDR samples. The results of all these findings will likely contribute to better management of DR samples in the future and hopefully provide a foundation for future research on the screening of new therapeutic targets for DR. In addition, our findings may provide clinicians with a new insight into making better treatment decisions for DR samples.

## Data availability statement

The original contributions presented in the study are included in the article/supplementary material. Further inquiries can be directed to the corresponding authors.

## Ethics statement

The studies involving human participants were reviewed and approved by The Ethical Committee of Peking University People’s Hospital (2021PHB112-001). The patients/participants provided their written informed consent to participate in this study.

## Author contributions

ZW drafted the work, revised it critically for important intellectual content, and provided approval for publication of the content. JT revised and reviewed the work critically for important intellectual content. EJ, CR and SL analyzed and interpreted data for the work. Material preparation and data collection were performed by LZ, YZ and YC. JW, WZ and MZ reviewed, edited and supervised the work. LH and JQ agreed to be accountable for all aspects of the work in ensuring that questions related to the accuracy or integrity of any part of the work are appropriately investigated and resolved. All authors contributed to the article and approved the submitted version.

## Funding

This study was supported by the Beijing-Tianjin- Hebei Special Project (Grant Number J200014), Capital clinical diagnosis and treatment technology research and demonstration application project of China (Grant Number Z191100006619029), National Key R&D Program (Grant Number 2020YFC2008200), Science and technology innovation project of Chinese academy of medical sciences (Grant Number 2019-RC-HL-019) and the National Natural Science Foundation of China (Grant Number 81670870). The funders had no role in the study design, data collection and analysis, decision to publish or preparation of the manuscript.

## Acknowledgments

We thank Dr. Huixin Liu, Department of Clinical Epidemiology and Biostatistics, Peking University People’s Hospital and Dr. Xueying Qin, Department of epidemiology and health statistics, School of public health, Peking University for the data analysis and instruction in statistics.

## Conflict of interest

The authors declare that the research was conducted in the absence of any commercial or financial relationships that could be construed as a potential conflict of interest.

## Publisher’s note

All claims expressed in this article are solely those of the authors and do not necessarily represent those of their affiliated organizations, or those of the publisher, the editors and the reviewers. Any product that may be evaluated in this article, or claim that may be made by its manufacturer, is not guaranteed or endorsed by the publisher.
